# Diagnostic accuracy of atopy patch test in children with cow’s milk allergy

**DOI:** 10.1186/s12948-023-00183-6

**Published:** 2023-04-07

**Authors:** Prapasri Kulalert, Padcha Pongcharoen, Paskorn Sritipsukho, Sukkrawan Intraakhao, Punnapat Piriyanon, Patcharapa Thaweekul, Sira Nanthapisal, Orapan Poachanukoon

**Affiliations:** 1 Department of Clinical Epidemiology, Faculty of Medicine, Thammasat University, Pathum Thani, 12120 Thailand; 2 Department of Pediatrics, Faculty of Medicine, Thammasat University, Pathum Thani, Thailand; 3 Center of Excellence in Applied Epidemiology, Thammasat University, Pathum Thani, Thailand; 4 Center of Excellence for Allergy, Asthma and Pulmonary Disease, Thammasat University, Pathum Thani, Thailand; 5 Department of Medicine, Faculty of Medicine, Thammasat University, Pathum Thani, Thailand

**Keywords:** Cow’s milk allergy, Atopy patch test, Diagnostic accuracy, Child

## Abstract

**Background:**

The accuracy of an atopy patch test (APT) for fresh cow’s milk allergy is controversial. Few studies have focused on commercial extract solutions. We aimed to evaluate the diagnostic performance of the APT in cow’s milk allergic children using fresh cow’s milk and commercial extracts of cow’s milk and its components including casein, α-lactalbumin, and β-lactoglobulin.

**Methods:**

A prospective study was carried out in children with a history of cow’s milk allergy. Children underwent the skin prick test (SPT) and APT with fresh cow’s milk, powdered cow’s milk, and commercial extracts of cow’s milk, casein, α-lactalbumin, and β-lactoglobulin. Oral food challenge (OFC) was confirmed in all children.

**Results:**

A total of 37 patients participated (mean age 13.14 ± 7.26 months). Only 5 (13.51%) patients had positive OFC to cow’s milk. The sensitivity of the APT using fresh cow’s milk was 40%, specificity was 65.6%, PPV was 15.4%, and NPV was 87.5%. The sensitivity of the APT using powdered cow’s milk was 40%, 60.7% for specificity, 15.4% for PPV, and 58% for NPV. The sensitivity and PPV of the APT using commercial solutions of cow’s milk, casein, α-lactalbumin, and β-lactoglobulin were zero. The specificities were 90.6%, 93.8%, 100%, and 100% for α-lactalbumin, cow’s milk, casein, and β-lactoglobulin, respectively.

**Conclusions:**

APT using commercial solutions showed higher specificity than fresh milk. The specificity increased using a protein component allergen.

## Introduction

The atopy patch test (APT) has been proposed as an investigation tool to assess children with clinically suspected non-IgE-mediated food allergy [1]. Cow’s milk allergy (CMA) is one of the common food allergies in children [2]. Symptoms of non-IgE-mediated CMA are mostly delayed reaction that occur beyond 2 h following ingestion and usually involve the gastrointestinal system (e.g., proctocolitis) or skin (e.g., dermatitis) or both [3, 4].

The APT with fresh cow’s milk has been investigated in children with atopic dermatitis and CMA related gastrointestinal symptoms. However, the utility for CMA diagnosis remains controversial [5–11]. In addition, the limitation is fresh food allergens spoil easily.

A liquid solution on filter paper can be used for APT [12, 13]. The commercial extract preparation is more stable than fresh allergens, and it is easy to prepare and perform in clinical practice. Cow’s milk solutions for skin testing are currently available. Furthermore, casein, α-lactalbumin, and β-lactoglobulin are major allergens in cow’s milk [14], which are available as commercial test solutions.

To the best of our knowledge, no study has evaluated the accuracy of the APT using commercial extracts of cow’s milk or the specific components of casein, α-lactalbumin, and β-lactoglobulin. Moreover, the APT with fresh cow’s milk has not been evaluated among Thai children.

The objective of this study was to evaluate the APT diagnostic performance in children with suspected CMA using fresh cow’s milk and commercial extracts for cow’s milk and its components including casein, α-lactalbumin, and β-lactoglobulin.

## Methods

### Study design and study patients

A prospective study was carried out at the Center of Excellence for Allergy, Thammasat Hospital, Pathum Thani, Thailand between January 2017 and December 2018. Children were enrolled in the study if they met all of the following criteria: (1) a history of at least one symptom such as dermatitis or mucous bloody diarrhea; (2) presented with delayed onset of CMA (i.e., symptom appeared > 2 h after ingestion); and (3) the symptoms improved when cow’s milk was eliminated.

Children with a history of suspected IgE-mediated CMA (e.g., anaphylaxis, urticaria, angioedema), or severe non-IgE-mediated CMA (e.g., food protein induced enterocolitis syndrome), or subjective symptoms (e.g., pruritus) were excluded. We also excluded children who did not complete the allergy testing.

The study was approved by The Institutional Review Board and the Ethics Committee of the Faculty of Medicine, Thammasat University. Informed consent was obtained from the parents of all children.

### Study protocol

All children underwent the skin prick test (SPT) and APT at the first visit. Reading of the APT results was performed at the second and third visits. At the fourth visit, the children underwent the open oral food challenge (OFC). If a reaction did not occur, the intake of cow’s milk continued at home.

### Skin prick test

All children were advised to discontinue oral antihistamine for 7 days before testing.

The SPT was carried out using 7 reagents: (1) fresh cow’s milk; (2) 1 g of powdered skim cow’s milk with 10 mL of isotonic saline solution as a vehicle; (3) commercial solution of cow’s milk; (4) commercial solution of casein; (5) casein reagent solution; (6) α-lactalbumin reagent solution; and (7) β-lactoglobulin reagent solution. Items 3 and 4 were obtained from ALK-Abelló (Port Washington, NY, USA) and items 5, 6, and 7 were provided by Lofarma S.p.A (Milan, Italy). Histamine 1 mg/mL and 50% glycerin were used as positive and negative controls, respectively.

One drop of each reagent was applied to the volar surface of the forearm using a 1 mm single peak lancet by experienced nurses and read after 15‒20 min. Each of the 7 reagents were given a code number to maintain blinding for interpretation of the results. The results were read by a nurse who was not involved with the APT or OFC test. Wheal size was measured by the longest and orthogonal diameters, reported as millimeters (mm). SPT positive was defined as a wheal diameter ≥ 3 mm than the negative control.

### Atopy patch test

All children were instructed not to apply topical corticosteroids or topical calcineurin to the test site for 5‒7 days before the test. They were also instructed not to put any creams or oils on their backs the morning of the testing [13].

The APT was carried out using one drop each of the 7 reagents. The solution was put on filter paper using 8 mm aluminum test cups (Finn Chambers on Scanpore; Epitest Ltd Oy, Tuusula, Finland). Vaseline was the negative control. The Finn Chambers were applied to the upper back and covered with non-allergenic plasters. All reagents were assigned a code number to maintain blinding. Evaluation took place 48 h after application, and the results were read 20 min after removing the Finn Chambers by dermatologists who were blinded to the SPT results and independent of the OFC testing. The second reading of the APT was performed 72 h post-application by the same dermatologist.

Reactions were classified according to the European Task Force on Atopic Dermatitis protocol as follows: –, negative, no reaction; ?, only erythema, questionable; + , erythema, infiltration; +  + erythema, few papules; +  +  + , erythema, many or spreading papules, and +  +  +  + erythema, vesicles [15].

### Open oral food challenge

The open OFC was performed 1 week after the APT was read. Before the OFC, each child underwent a physical examination. The OFC was performed only when the child was asymptomatic and had a completely normal physical examination. Emergency medications, such as epinephrine, antihistamine, and steroids, and equipment were prepared.

On day 1, the OFC was performed at the hospital. The protocol was applied based on a previous consensus report [16]. Since our cases were at low risk of developing a severe acute reaction, we omitted 3, 10, and 30 mg. The initial dose started with 100 mg of cow’s milk protein. The OFC was performed by administering increasing doses (100, 300, 1000, 3000, 4000 mg) at 20 min intervals with the total amount of cow’s milk protein being 8400 mg [17].

During the OFC the children were monitored for vital signs, abnormal signs, and symptoms (e.g., skin rash, vomiting) before escalating each dose. Challenges were discontinued if they developed clinical symptoms. Patients were observed for a minimum of 4 h after the last dose and if no reaction occurred, they were discharged. The OFC was performed and assessed by an experienced nurse and pediatric allergist who were blinded both of the SPT and APT results.

On days 2‒7, the children continued to receive cow’s milk formula with full servings of 6‒8 oz at home. The parents were advised to observe for any symptomatic reaction to the milk [17]. If any clinical symptoms occurred during this time, the parents were told to contact and visit our clinic as soon as possible. On the seventh day and 1 month after the OFC the children were followed up and observed for any delayed reaction.

OFC was defined as positive if a child had at least one of the following clinical symptoms: vomiting; diarrhea; hematochezia; or dermatitis. Immediate reactions were defined as those occurring within 2 h after the last dose of OFC and delayed reactions were defined as reactions observed after 2 h of the last dose.

### Statistical analysis

The statistical analysis was performed using STATA version 14.0. Demographic characteristics were described as mean ± standard deviation for continuous variables as appropriate. Frequency was used for categorical variables. APT accuracy was calculated in terms of sensitivity, specificity, positive predictive value (PPV), and negative predictive value (NPV).

## Results

A total of 37 children participated and completed the study. There were 19 (51.35%) girls and 18 (48.65%) boys, and the mean age was 13.14 ± 7.26 months. Twenty-five (67.57%) patients had dermatitis, 12 (32.43%) had mucous bloody stool, 3 (8.11%) reported vomiting, and 3 (8.11%) reported watery diarrhea after cow’s milk ingestion. From the 37 children, only 5 (13.51%) had positive OFC results for cow’s milk (Table 1).

**Table 1:** Characteristics of the study population

Characteristics	
Mean age (months), mean ± SD	13.14 ± 7.26
Gender	
Female	19 (51.35)
Male	18 (48.65)
Clinical manifestations^a^	
Dermatitis	25 (67.57)
Gastrointestinal symptoms	
Mucous bloody stool	12 (32.43)
Vomiting	3 (8.11)
Watery diarrhea	3 (8.11)
Oral cow’s milk challenge	
Positive	5 (13.51)
Immediate reaction	3 (8.11)
Delayed reaction	2 (5.40)
Negative	32 (86.49)

Data are presented as n (%) unless otherwise indicated

*SD* standard deviation

^a^some cases had > 1 symptom

The SPT and APT results are summarized in Table 2 and the diagnostic accuracy of the SPT and APT are reported in Table 3. All children were negative for casein and α-lactalbumin. Four children showed positive to fresh and powdered skim milk, 3 children were positive for β-lactoglobulin, and 1 child was positive for cow’s milk (ALK-Abelló) solution. However, these 4 children were false positive since they passed the OFC to cow’s milk.

**Table 2:** Results of skin prick test and atopy patch test (*N* = 37)

	Positive	True positive^a^	False positive^b^	Negative	True negative^c^	False negative^d^
Skin prick test						
Fresh	4	0	4	33	28	5
Powder skimmed milk, (N = 33)	4	0	4	29	24	5
Cow’s milk (ALK-Abelló)	1	0	1	36	31	5
Casein (ALK-Abelló)	0	0	0	37	32	5
Casein (Lofarma)	0	0	0	37	32	5
α-lactalbumin (Lofarma)	0	0	0	37	32	5
β-lactoglobulin (Lofarma)	3	0	3	34	29	5
Atopy patch test						
Fresh	13	2	11	24	21	3
Powder skimmed milk, (*N* = 33)	13	2	11	20	17	3
Cow’s milk (ALK-Abelló)	2	0	2	35	30	5
Casein (ALK-Abelló)	1	0	1	36	31	5
Casein (Lofarma)	0	0	0	37	32	5
α-lactalbumin (Lofarma)	3	0	3	34	29	5
β-lactoglobulin (Lofarma)	0	0	0	37	32	5

^a^True positive (+ APT, + OFC), ^b^false positive (+ APT, − OFC), ^c^true negative (− APT, − OFC), and ^d^false negative (− APT, + OFC)

**Table 3:** Diagnostic performance of skin prick test and atopy patch test

	Sensitivity (%)	Specificity (%)	PPV (%)	NPV (%)
Skin prick test				
Fresh	0	87.5	0	84.8
Powder skimmed milk	0	85.7	0	82.8
Cow’s milk (ALK-Abelló)	0	96.9	0	86.1
Casein (ALK-Abelló)	0	100	0	86.5
Casein (Lofarma)	0	100	0	86.5
α-lactalbumin (Lofarma)	0	100	0	86.5
β-lactoglobulin (Lofarma)	0	90.6	0	85.3
Atopy patch test				
Fresh	40	65.6	15.4	87.5
Powder skimmed milk	40	60.7	15.4	85.0
Cow’s milk (ALK-Abelló)	0	93.8	0	85.7
Casein (ALK-Abelló)	0	96.9	0	86.1
Casein (Lofarma)	0	100	0	86.5
α-lactalbumin (Lofarma)	0	90.6	0	85.3
β-lactoglobulin (Lofarma)	0	100	0	86.5
Skin prick test + Atopy patch test (combination test)^a^				
Fresh	0	96.9	0	86.1
Powder skimmed milk	0	92.9	0	83.9
Cow’s milk (ALK-Abelló)	0	100	0	86.5
Casein (ALK-Abelló)	0	100	0	86.5
Casein (Lofarma)	0	100	0	86.5
α-lactalbumin (Lofarma)	0	100	0	86.5
β-lactoglobulin (Lofarma)	0	100	0	86.5

*PPV* positive predictive value, *NPV* negative predictive value

^a^Test positive was defined as positive to SPT and APT

From the total of 37 patients, 13 (35.14%) patients showed positive APT to fresh milk. Eleven of these 13 children had + APT results, which were determined to be clinically irrelevant as they did not experience problems during the OFC. However, 2 of the 13 children with positive APT developed reactions during the OFC. Among the 24 children who had − APT, 21 children could actually tolerate cow’s milk. However, 3 children appeared to have had false negative to APT as they developed reactions during the OFC. The sensitivity was 40%, specificity was 65.6%, PPV was 15.4%, and NPV was 87.5%.

Of the 33 children who underwent the APT with powdered skim milk, 13 (39.4%) children showed + APT to powdered skim milk. Eleven of these 13 children who had + APT had results that were determined to be clinically irrelevant as they did not experience problems during the OFC; however, 2 of the children with + APT developed reactions during the OFC. Among the 20 children who had − APT results, 17 could actually tolerate cow’s milk. However, 3 appeared to have had false negative to APT as they developed reactions during the OFC. The APT using powdered skim milk had diagnostic accuracies of sensitivity 40%, specificity 60.7%, PPV 15.4%, and NPV 85%.

Two children had + APT to the commercial solutions of cow’s milk extracts. However, none of them had a reaction after consuming cow’s milk. The sensitivity and PPV were equal to zero, while the specificity and NPV were 93.8% and 85.7%, respectively.

Three children showed false positive results to the cow’s milk component of α-lactalbumin, and one child showed false positive to casein (ALK-Abelló). All children in our study were negative to casein (Loforma) and β-lactoglobulin. The sensitivity and PPV were equal to zero for the three cow’s milk components. The specificity was 90.6% for α-lactalbumin, 96‒100% for casein, and 100% for β-lactoglobulin.

The performance of combining the SPT and APT is shown in Table 3. The test positive was defined as positive to SPT and APT. Combining APT with SPT improved the diagnostic performance of the specificity compared to the SPT or APT alone.

## Discussion

In this study, we aimed to demonstrate the accuracy of the APT with fresh milk, commercial extracts of cow’s milk, casein, α-lactalbumin, and β-lactoglobulin in children with clinical suspicion of non-IgE-mediated CMA.

The sensitivity was 40% for fresh milk, which was consistent with previous studies. Roehr et al. reported that the sensitivity of the APT was 47% and Mehl et al. reported a sensitivity of 37% [18, 19]. Moreover, our sensitivity results were in accord with a meta-analysis by Lou et al. who reported that the pooled sensitivity of the APT for cow’s milk was 44.2% (95% confidence interval (CI) 41.5‒47.0%) [20].

We showed the APT to have low specificity (65.6%) for fresh milk. This was less than other studies that tested with fresh milk and reported specificities of 95‒96%, and a meta-analysis showed a specificity of 86.9% (95% CI 85.0‒88.7%) [18–20].

The low specificity may be associated with the high false positive rate. False positive was defined as patients who were + APT but demonstrated tolerability to cow’s milk ingestion; therefore, the results in those patients were not clinically relevant. Our study showed a high rate of false positive for fresh milk.

False positive reactions in the APT test can result from hyperirritability of the skin due to irritants or allergic substances or patch testing on skin with active dermatitis [13]. Therefore, we attempted to minimize the chances for false positives by using the Finn Chamber with the built-in hypoallergenic adhesive, which was in line with other studies. Vaseline was our negative control to exclude false positive reactions. All children had normal skin appearance before the APT test.

It must be noted that some of our false positives from an irritant extract were possibly due to environmental conditions. Thailand is in a tropical region with year-round humidity with rapid food spoilage, especially for dairy products. It is likely some of our false positives were due to irritation from environmental factors, not from true allergies.

Moreover, our study also showed low specificity and high false positive for powdered skim milk diluted in isotonic saline solution as a vehicle. Those results support our idea that the APT with fresh food should be performed with caution in tropical and humid countries.

In this current study, the APT using powdered skim milk had a sensitivity that was near a previous study but with a lower specificity than that study [21]. Gonzaga et al. performed the APT using skimmed cow milk powder in a saline solution in 32 children with non-IgE-mediated CMA. The results were 33% for sensitivity and 96% for specificity.

The accuracy of the APT using the commercially available cow’s milk solution had a low sensitivity but a high specificity of 93.8%. The result was similar to a previous study also performed in Thailand. The specificity of the APT with commercial allergen extracts was 90% [22].

Recently, casein, α-lactalbumin, and β-lactoglobulin were identified as major allergens in cow’s milk. Sozmen et al. performed the ATP using lyophilized cow’s milk and casein in white Vaseline as the excipient. The specificity (80%) was higher than the sensitivity (15%) [23].

The skin test solutions of these three specific allergens are available; however, there is a lack of in vivo evidence for testing in children with a non-IgE-mediated reaction. We found that the APT using casein, α-lactalbumin, and β-lactoglobulin commercial extracts gave high specificities in the range of 90‒100% but low sensitivities, which was similar to previous studies using lyophilized food in white Vaseline as the excipient.

When we compared the accuracies between fresh food and the commercial solutions, fresh food was found to have greater sensitivity than the commercial extracts. Similarly, a previous study that used commercial extracts had a sensitivity of only 6% but the specificity was 95% [6].

The combination of SPT and APT has produced controversial results [18, 19]. We demonstrated that combining APT with SPT improved the specificity compared to SPT or APT alone.

The specificity of a test (also called the true negative rate) is the proportion of people without disease who will have negative test results. In other words, the specificity of a test refers to how well a test identifies patients who do not have a disease. A test that has high specificity will identify a high proportion of patients who do not have the disease. In our study, a high specificity of the APT may be useful to identify children without reactions to the OFC test. Thus, the APT using the commercial allergen solutions, especially the component proteins of casein and β-lactoglobulin, may be useful for clinicians to encourage further OFC in children if they are negative to the APT, which would help decrease unnecessary elimination diets.

The results of this study indicate that using commercial extracts in the APT may be more appropriate than fresh food in humid countries as evidenced by the higher specificity and lower false positive results.

The strength of our study was the well-designed research methodology to prevent information bias. Our APT interpretation was performed by a dermatologist who was independent and blinded for comparison with the OFC testing. The nurses and allergists who performed the OFC did not know the APT results.

However, this study had limitations. First, our OFC was open. A double-blind, placebo-controlled OFC is the gold standard for the diagnosis of food allergies; however, the open method is easier to perform in practice. The open OFC has been used more often, especially among children up to 3 years of age [24]. In this study, the children were younger than 3 years of age (mean age 13.14 ± 7.26 months). The second limitation was the small sample size and low proportion of patients who had positive OFC results for cow’s milk. A further study is needed with a larger sample size that should consider using a diagnostic accuracy study design in low prevalence situations (e.g., a two-gate case-control design) [25]. The third limitation was we did not perform a subgroup analysis based on dermatitis or gastrointestinal symptoms.

In conclusion, the APT with commercial solutions showed high specificity in evaluating children with CMA. In addition, the specificity increased using component allergens of cow’s milk. Even though the APT is a very specific test, its low sensitivity might hinder its use in clinical practice. The APT with fresh food is not a valid tool in humid countries to diagnose CMA in children due to false positive results that occur.

## Data Availability

The dataset analyzed in the current study is available from the corresponding author on reasonable request.

## Abbreviations

SPT: Skin prick test
APT: Atopy patch
OFC: Oral food challenge
